# Commentary: Current Status of Gene Therapy for Spinal Muscular Atrophy

**DOI:** 10.3389/fncel.2022.916065

**Published:** 2022-05-17

**Authors:** Wilfried Rossoll, Ravindra N. Singh

**Affiliations:** ^1^Department of Neuroscience, Mayo Clinic, Jacksonville, FL, United States; ^2^Department of Biomedical Sciences, Iowa State University, Ames, IA, United States

**Keywords:** spinal muscular atrophy (SMA), Survival Motor Neuron (SMN), onasemnogene abeparvovec, Zolgensma, AVXS-101, gene therapy, AAV9

## Introduction

Spinal muscular atrophy (SMA) is a genetic disease of a broad spectrum of severity, ranging from infant mortality to adult onset (Singh et al., 2021). SMA results from low levels of Survival Motor Neuron (SMN) protein due to deletions or mutations of the *SMN1* gene (Wirth et al., 2020). *SMN2*, a nearly identical copy of the *SMN1* gene, fails to compensate for the loss of *SMN1* due to a single nucleotide change in a splice site that causes predominant skipping of exon 7 (Wirth et al., 2020). Correction of *SMN2* exon 7 splicing by nusinersen, an antisense oligonucleotide (ASO), became the first approved therapy for SMA in 2016 (Singh N. N. et al., 2017). Onasemnogene (Synonyms: Onasemnogene abeparvovec, Zolgensma, AVXS-101), an adeno-associated virus serotype 9 (AAV9)-mediated gene therapy was approved in 2019 for the treatment of SMA (Al-Zaidy et al., 2019). Risdiplam was approved in 2020 as the first orally deliverable small molecule therapy for SMA (Singh et al., 2020b). These developments constitute unparalleled success for the treatment of an orphan disease. Like nusinersen, risdiplam promotes exon 7 inclusion from the endogenous *SMN2* gene, which is universally present in SMA patients (Singh N. N. et al., 2017; Singh et al., 2020a). However, unlike nusinersen that is administered intrathecally at the interval of several months, daily oral administration of risdiplam is required for the intended therapeutic benefits. Gene therapy, on the other hand, does not depend upon the availability of endogenous *SMN2* and is conceptually suited to provide the sustained source of SMN upon a single systemic administration (Al-Zaidy et al., 2019). A general challenge for SMA therapy is the irreparable loss of motor units that cannot be restored due to delayed administration of the drug and has led to the addition of SMA to the Recommended Uniform Screening Panel (RUSP) for newborn screening programs. Here we discuss recent reports underscoring the challenges associated with gene therapy. We also propose strategies to improve the future gene therapy-based approaches for an effective and risk-free treatment of SMA.

## Challenges Associated With Gene Therapy of SMA

Specific challenges associated with gene therapy include preparation and administration of the large therapeutic molecule (packaged AAV9 vector containing transcription-competent *SMN* cDNA) and efficient body-wide distribution of the delivered gene, including the central nervous system (CNS). Overexpression of the delivered gene and the inflammatory response against the AAV9 vector itself are the additional concerns of gene therapy. While neuronal tissues are known to be highly susceptible to low SMN levels in severe SMA, accumulating evidence supports widespread developmental defects during the embryonic stages of severe SMA (Motyl et al., 2020). Abnormalities in peripheral tissues manifest more commonly in longer surviving milder SMA patients (Singh et al., 2021). SMA patients and SMA mouse models display abnormal glucose, lipid and amino acid metabolisms, although the magnitude of abnormalities differs with respect to the type of SMA and the patient's age (Singh et al., 2021). In addition, SMA patients have increased susceptibility to dyslipidemia, liver steatosis and non-alcoholic fatty acid disease (Singh et al., 2021). With the ongoing treatments, severe SMA cases that used to cause early lethality may be transformed into milder SMA cases of a chronic disease with peripheral defects remaining to be addressed.

Several recent studies are trying to address gaps in our knowledge about SMN expression levels and their consequences. A recent report conducted on a mouse model of SMA confirms the lifelong need of SMN in all tissues, including peripheral tissues (Zhao et al., 2021). The first study performed on the postmortem tissues of SMA patients treated with gene therapy showed body-wide delivery of onasemnogene (Thomsen et al., 2021). However, distribution was uneven based on the counts of the AAV9 copy number per diploid genome. The study was conducted with only two SMA patient samples and result showed patient to patient variability. One of the patients had degraded RNA that could not be reliably used for determining the *SMN* transcripts generated from the onasemnogene (Thomsen et al., 2021). Based on the experiments conducted on a single onasemnogene treated patient sample, authors found non-uniform expression of *SMN* transcripts in different tissues (Thomsen et al., 2021). Further, the study employed as a reference for normalization *GAPDH* that showed drastic variability in expression among different tissues. Motor neurons of the onasemnogene treated patient showed comparable size and shape to that of the non-SMA control spinal motor neurons (Thomsen et al., 2021). Histological analysis of peripheral tissues, including heart, intestine, kidney, liver, lung, lymph nodes, muscle, pancreas, spleen and thymus showed expression of SMN protein (Thomsen et al., 2021). As a caveat, since both SMA patients died within few months of the onasemnogene treatment, findings may be representative of unsuccessful cases of gene therapy and may not represent typical distribution and efficacy of onasemnogene. Despite these inherent limitations, this study indicates widespread expression of SMN throughout the CNS after intravenous administration of onasemnogene in humans.

Recent reports on outcome of three clinical trials of onasemnogene on SMA patients of ages younger than 6 months to 2 years showed mixed results (Day et al., 2021; Mercuri et al., 2021; Weiß et al., 2022). Statistically significant benefits of onasemnogene treatment were noted in most SMA patients (Day et al., 2021; Mercuri et al., 2021; Weiß et al., 2022). Notably, patients previously treated with nusinersen also benefited from gene therapy (Weiß et al., 2022). However, findings revealed several treatment-related adverse events. For instance, pyrexia was the universal adverse event in all three clinical trials with onasemnogene (Day et al., 2021; Weiß et al., 2022). Additional adverse events included bronchiolitis, pneumonia, respiratory distress, and respiratory syncytial virus bronchiolitis in younger SMA patients (Day et al., 2021). In a previous report, onasemnogene treated patients showed abnormally high levels of *SMN* expression in the liver due to the known hepatotropism of AAV9 vector (Thomsen et al., 2021). Consistently, immunological assays confirmed an immune response against AAV9 capsid proteins (Thomsen et al., 2021). While inflammatory response of liver could be mitigated to certain extent by prednisolone, continued accumulation of *SMN* transcripts and protein in liver remain a concern in the long run (Thomsen et al., 2021; Weiß et al., 2022). Peripheral tissues, including intercostal muscle, diaphragm and pancreas of SMA patients treated with onasemnogene also showed abnormal high expression of *SMN* transcripts, raising concerns similar to those noted with liver (Thomsen et al., 2021).

It is too soon to make any definitive conclusion about the long-term effects of gene therapy. However, a previous study on non-human primates and piglets following IV administration of an AAV9 variant encoding SMN, and a more recent study conducted in a severe mouse model of SMA is instructive for understanding potential long-term consequence of the AAV9-mediated gene therapy of SMA (Hinderer et al., 2018; Van Alstyne et al., 2021). In particular, the mouse study found synaptic loss and motor neurodegeneration due to toxicity of the overexpressed human SMN from AAV9-mediated gene therapy (Van Alstyne et al., 2021). Levels of endogenous proteins in a cell are modulated by an intricate network of feedback interactions that spans from transcriptional to post-transcriptional, translational and post-translational regulations but fail to limit the expression of exogenous cDNA expression cassettes. Abnormal overexpression of a protein comes at the expense of the synthesis of other proteins, given the limited supply of the building blocks of the protein synthesizing machinery, including ribosomes, tRNAs and amino acids. Effect of overexpression is further compounded due to unwanted sequestering of proteins and RNAs interacting with both the overexpressed transcript and protein. Toxic effect of SMN overexpression is particularly expected based on the fact that SMN is an RNA binding protein and also interacts with a host of proteins associated with the regulation of essential cellular processes, including DNA replication and repair, transcription, pre-mRNA splicing, translation, stress granule formation, macromolecular trafficking, signal transduction and cytoskeletal dynamics (Singh R. N. et al., 2017; Price et al., 2018). However, findings of toxicity of overexpressed SMN in the mouse model should be interpreted with caution because a different promoter was used in the AAV9 vector to express *SMN*. Furthermore, unlike the intrathecal route of delivery in the mouse study, SMA patients receive onasemnogene through intravenous administration (Al-Zaidy et al., 2019; Mercuri et al., 2021; Weiß et al., 2022). In addition, gene delivery in the mouse study was performed at postnatal day 0 when the blood brain barrier is not completely formed (Van Alstyne et al., 2021). Supporting these caveats, postmortem neuronal tissues of SMA patients intravenously treated with onasemnogene did not show *SMN* overexpression in neuronal tissues (Thomsen et al., 2021).

## Concluding Remarks

**Figure 1:**
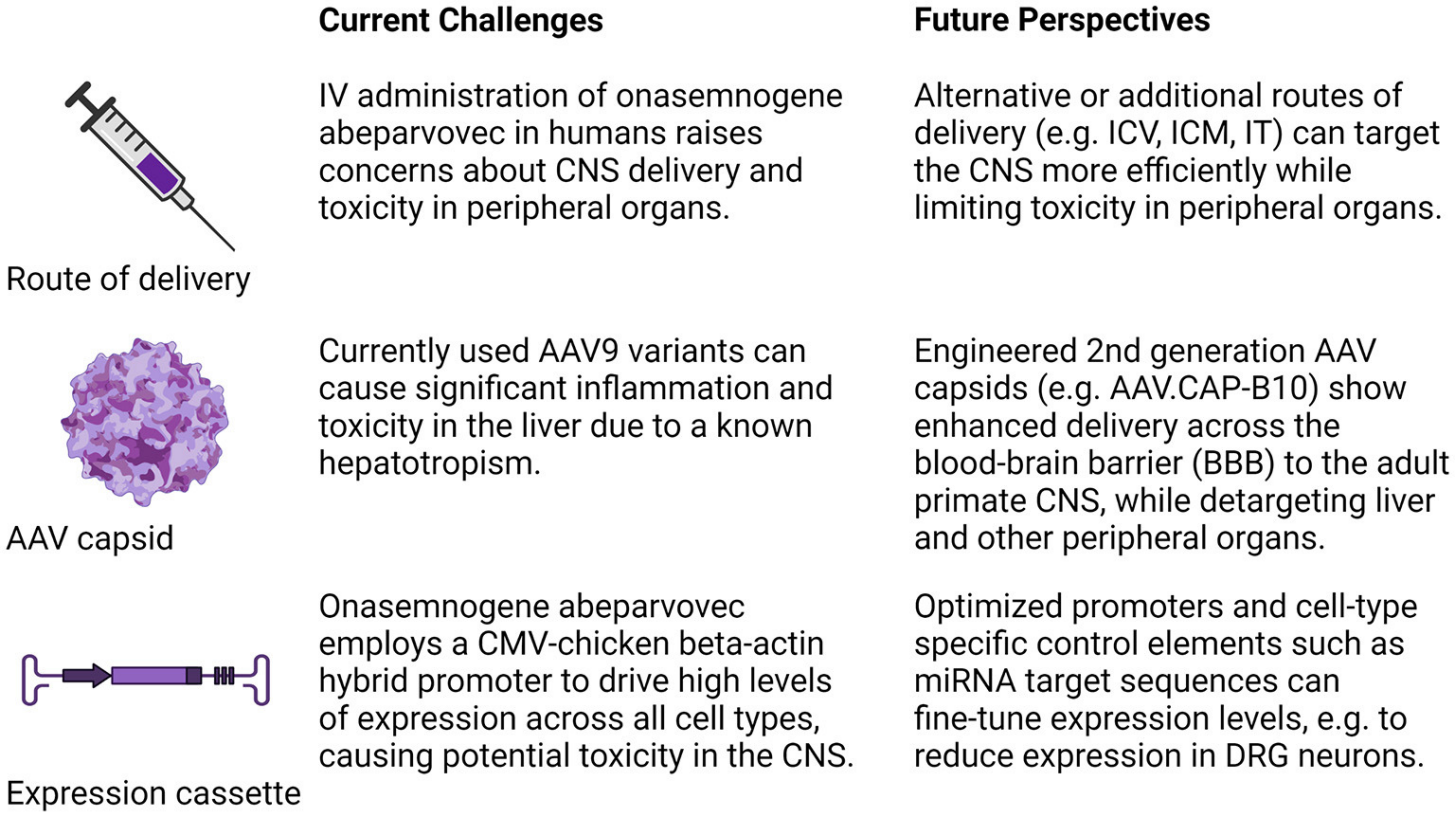
Strategies for increasing therapeutic transduction efficiency and reducing toxicity. Concerns raised by recent studies and future strategies to address these challenges as they relate to the route of administration, the AAV serotype/capsid, and expression construct are summarized. IV, intravenous; ICV, intracerebroventricular; ICM, intracisterna magna, IT, intrathecal; CMV, cytomegalovirus; miRNA, micro-RNA. Figure created with BioRender.com.

In conclusion, studies thus far highlight the need to develop the next generation of gene therapy with an improved body-wide distribution and embedded control elements that limit expression levels and on/off switches so that *SMN* expression can be finetuned. Potential strategies for increasing CNS delivery and selectivity are outlined in Figure 1. They include different methods of administering/delivering the virus to the target tissue (IV, ICV, ICM, IT) and the engineering of a new generation of AAV capsids with enhanced delivery across the blood-brain barrier (BBB) to the adult primate CNS, while detargeting liver and other peripheral organs (Fischell and Fishman, 2021). Selecting optimized promoters and other tissue and cell-type specific control elements such as miRNA target sequences can provide an additional level of selectivity for finetuning the expression in transduced cells (Hordeaux et al., 2020). In addition, these studies also highlight the need for long-term animal safety research and continued basic research to provide a better understanding of toxicity associated with the prolonged SMN overexpression. Given the indication that males are disproportionally impacted in SMA (Singh et al., 2021; Zhao et al., 2021), it would be important to assess if the long-term effect of gene therapy has any gender-specific bias. In principle, gene therapy has the potential to deliver the best therapeutic outcome upon single administration. However, the task of achieving that goal would depend upon the pace with which deficiencies of the current approach are recognized and rectified.

## Author Contributions

WR and RS conceived idea, analyzed data, and reviewed the literature and wrote the manuscript. All authors contributed to the article and approved the submitted version.

## Funding

RS was supported by grants from NIH (R01 NS055925), a recipient of the 2006 Presidential Early Career Award for Scientists and Engineers (PECASE) for the discovery of the ISS-N1 target (US7838657) which led to the development of nusinersen (Singh et al., 2020b), the first FDA-approved drug of SMA, receives royalty payments for ISS-N1 discovery and could potentially benefit from other related inventions, and serves as a consultant to Pharmaceutical companies engaged in drug development. WR was supported by grants from the NIH (R33 NS110960) and DoD (W81XWH-19-1-0193). Open access publication support was provided by Iowa State University Library, Ames, Iowa.

## Conflict of Interest

The authors declare that the research was conducted in the absence of any commercial or financial relationships that could be construed as a potential conflict of interest.

## Publisher's Note

All claims expressed in this article are solely those of the authors and do not necessarily represent those of their affiliated organizations, or those of the publisher, the editors and the reviewers. Any product that may be evaluated in this article, or claim that may be made by its manufacturer, is not guaranteed or endorsed by the publisher.
